# Comparative evaluation of convolutional and transformer-based object detection models for brain tumor localization in MRI

**DOI:** 10.1371/journal.pone.0358483

**Published:** 2026-09-24

**Authors:** Farhan Naeem, Amara Haroon, Nasru Minallah, Tufail Ahmad, Shahi Dost

**Affiliations:** 1 Department of Computer Systems Engineering, University of Engineering and Technology, Peshawar, Pakistan; 2 Department of Radiology, Institute of Kidney Diseases Hayatabad Medical Complex, Peshawar, Pakistan; 3 Department of Computer Systems Engineering, University of Engineering and Technology, Peshawar, Pakistan; 4 National Center for Bigdata and Cloud Computing, University of Engineering and Technology, Peshawar, Pakistan; 5 Department of Mathematical Sciences, University of Liverpool, Liverpool, United Kingdom; Northeastern University, CHINA

## Abstract

There is a need to detect and precisely localize brain tumors based on medical imaging to ensure such tumors are diagnosed early and treated with the aim of a successful treatment. More recent developments in the field of deep learning have presented convolution-based and transformer-based object detection models that can be used to achieve high detection rates. We carry out an analogous comparison of YOLOv11s and RF-DETR(small) in brain tumor detection on annotated Magnetic Resonance Imaging (MRI) data on Roboflow in this study. Precision, Recall and mean Average Precision (mAP) along with localization loss measures were used to train and test both models. Findings indicate that YOLOv11s performed better in terms of mAP@50 of 95.2 percent, precision of 93.6 percent, and recall of 91 percent compared to RF-DETR that reported mAP@50 of 94.3 percent, precision of 90.6 percent, and recall of 85 percent. The findings point to the improved performance of YOLOv11s to localize and classify tumor locations in varying MRI slices. YOLOv11s with less convergent dynamic training behavior and RF-DETR(small) with better localization based on more challenging Intersection over Union (IoU) thresholds. The primary challenge was ensuring a fair comparison between two fundamentally different detection paradigms that employ distinct feature-learning mechanisms, pretraining schemes, optimization settings, and computational characteristics. Despite these, our findings highlight the feasibility of integrating advanced object detection models into computer-aided diagnostic tools for robust brain tumor detection in complex MRI data.

## Introduction

Brain tumors are the irregular growths of cells in the brain or the central nervous system that may interfere with vital nervous functions. They are categorized as benign and malignant, where the latter are very harmful as they tend to invade neighboring tissues and are also aggressive in nature, making them a major risk factor [1,2]. The early and precise diagnosis of these tumors is very important in enhancing survival and the therapeutic interventions. The most commonly used non-invasive diagnostic method to detect brain tumors has been Magnetic Resonance Imaging (MRI), which has excellent soft tissue contrast and structural resolution [3]. Nonetheless, manual interpretation of radiologist reviewed MRI images is lengthy, subjective, and susceptible to intra- and inter-observer errors, especially in situations with large volumes of imaging or tumor margins that are hard to detect [4,5].

As the Artificial Intelligence (AI) and Deep Learning (DL) has been growing exponentially, automated systems are being designed and created to assist radiologists in diagnostic procedures more and more frequently [6]. Convolutional neural networks (CNNs) have shown remarkable achievements in the medical image classification, segmentation, and detection of objects in the past few years [7]. Compared to the conventional methods of image processing based on hand-designed features, CNNs directly learn hierarchical and discriminative representations, which enhances the robustness of features and also generalization. AI integration in medical imaging is able to not only decrease the workload on diagnostics, but also improve the accuracy and repeatability of clinical decision-making. Object detection models are the types of deep learning that have become efficient in performing the activities that involve both classification and localization. Combining speed and precision, models like You Only Look Once (YOLOv11) [8] and Detection TRansformers (DETR) [9] have made a significant contribution to the field. YOLO has gone through multiple iterations into more advanced YOLOv11 which has incorporated architectural updates like Cross Stage Partial (CSP) connections, Transformer-based backbones, Decoupled Heads, and Spatial Pyramid Pooling. These adjustments have enhanced the accuracy of object localization significantly and at the same time, there is a preserved real-time performance. Conversely, RF-DETR (Real-time Fully DETR) is a transformer-based detector that uses the self-attention mechanism to learn global contextual connections in the images. RF-DETR allows a complete comprehension of image content unlike CNN-based architectures that pays more attention to the notion of local receptive fields, which enhances the object detection capabilities of the model, particularly regarding irregularly shaped objects or those that have diffused edges, which is a typical feature of medical images like brain tumors.

In spite of these developments, there are still issues related to the generation of reliable and clinically acceptable brain tumor detection. Most of the current models are affected by overfitting because of the scarcity of annotated data or cannot be extrapolated effectively across MRI images recorded using different protocols and using different devices. Additionally, CNNs are effective in the spherical features extraction, but they can hardly handle complex structural variations, whereas the transformer-based models consume large amounts of computing power. Therefore, to find the best compromise between accuracy, speed, and feasibility in a clinical environment, the comparative analysis of the two architectures is necessary. The proposed study involved a comparative deep learning architecture based on YOLOv11s and RF-DETR (Small) to detect brain tumors with the use of MRI scans. The aim is to evaluate the effectiveness of these models measured by Precision, Recall, Mean Average Precision (mAP@.5, mAP@.5:95), based on a high quality Brain Tumor MRI Dataset from Roboflow of 8,791 annotated images. The dataset consists of four classes, i.e., glioma, meningioma, pituitary, and no-tumor, whose bounding box annotations provide accurate training and validation on a per-region basis. The key challenge faced by this study is fair comparison between different object detection paradigms. CNN-based detectors that emphasize on efficient local feature extraction and real-time performance and transformer-based detectors, that rely on global attention mechanisms and object-query representations to capture long-range contextual information. The architectural differences are systematically assessed on common dataset and pre-training strategies, different resolution images and convergence characteristics etc.

A key limitation of this study is their dependence on single public datasets and the absence of external clinical validation, which may limit the generalizability of the findings. Moreover, most approaches rely exclusively on imaging information without incorporating additional clinical or pathological data that could further improve diagnostic performance.

The remaining part of the study is structured as; Literature Review which covers the related research work concerning this field, Material and Methods which reveals how this study was carried out. Data Pre-processing that displays methods used to prepare data to be used in model training. Model training and evaluation parameters are presented in the Model Training section and Results which displays performance evaluation of both the models.

### Literature review

The current developments in deep learning have optimally enhanced the detection and classification of brain tumors in MRI images. You Only Look Once (YOLO) family and transformer-based frameworks have become popular object detection methods to improve the localization accuracy and diagnostic efficiency.

Almufareh et al. [10] offered an automated brain tumor segmentation and classification framework based on YOLOv5 and YOLOv7, in which mask-alignment preprocessing approach was adopted to improve the accuracy of tumor localization of different classes of meningioma, glioma, and the pituitary tumor. Their models reported high detection performances of mAP@.5 of up to 0.947 with the potential of the application of YOLO-based segmentation in medical imaging. Nevertheless, they only studied older versions of YOLO and smaller datasets.

Based on this, Vinayakan et al. [11] introduced a transfer learning-based YOLO model that detects brain tumors using MRI, which successfully localizes tumours in various types but does not compare it with other architectures and has not analysed metrics in detail. The current study builds upon these studies, where the most recent YOLOv11 model is used and compared with the transformer-based RF-DETR model, thereby filling the gaps concerning scalability and model optimization.

In addition, Satheesh Kumar et al. [12] investigated the use of YOLOv11 to detect and classify brain tumors based on the data on MRI with data augmentation and transfer learning methods. Their model was highly accurate in differentiating between tumor and non-tumor cases, but was rather more oriented on the classification and not specific segmentation. By contrast, the present paper incorporates YOLOv11 with another model to facilitate fine-grained recognition and multi-stage feature acquisition to enhance the localization accuracy.

Previous studies by Montalbo [13] used a lightweight YOLOv4-Tiny model with transfer learning on 3,064 MRI images with 93.14% mAP, 90.34% precision and 88.58% recall. The model had limited computational effectiveness, but its shallow architecture restricted its segmentation and detection accuracy. The present paper mitigates this weakness by the superior transformer-based layers of YOLOv11 and focusing attention modules to provide more accuracy on a more extensive dataset of 8,791 MRI images.

Anjum Ali et al. [14] compared the performance of the YOLOv9, YOLOv10, and YOLOv11 at detecting brain tumor tasks with revealed that the YOLOv10 is more balanced in its performance with 0.95 precision and 0.88 recall, and that the YOLOv11 has a better real-time localization performance. Nevertheless, their data have only 1,003 images.

Equally, Alhussainan et al. [15] performed a comparative study on the meningioma firmness classification using YOLOv3 and YOLOv7 and obtained superior performances of 99.96% mAP and 99.24% F1 score. Although impressive, they focused on one aspect, which is firmness detection of meningioma.

Rahimi et al. [16] suggested a YOLOv4-Tiny-based model with the use of Gaussian and Sobel filters to enhance the visibility of tumor boundaries with a resulting mAP of 0.83. Their model could work but this was constrained by the capacity of lightweight architecture. In addition, to optimize YOLOv11n, Chen et al. [17] added GhostConv, OREPA, and Efficient Multiscale Attention (EMA) modules to obtain a 97.2% mAP and 93.8% recall with only 25 percent less computational complexity. They concentrated on the efficiency of architecture.

Raju et al. [18] further developed YOLOv11 into TwinFormer + Spatial-Semantic Feature Fusion with mAP50 of 0.950, precision of 0.936, and recall of 0.927 on BR35H and Real Brain Tumor datasets, by adding a TwinFormer and Segmented Cross-Flow Stage. They already showed that they can detect small tumors by offering transformer-based attention and could be used to enhance the process in the future with additional adaptability of features and attention.

Abdusalamov et al. [19] trained YOLOv7 with attention-based CBAM, SPPF + , and BiFPN modules with an outstanding result of 99.5% with a dataset of more than 10,000 MRI scans. Although it was very accurate, the model was not able to handle small and low-contrast tumours. Lastly, Jeyaraj and Kumar [20] came up with a hybrid YOLOv11-SAM segmentation framework that combines CNN and Vision Transformer modules into a complete system capable of extracting the detailed tumor boundaries with a mAP of 96.5%. Their method gave high accuracy in the segmentation masks, but it was very time consuming.

All these studies, however, demonstrate the extent of development in brain tumor detection research i-e the transition between conventional CNNs and first versions of YOLO to hybrid and transformer-augmented versions. Nevertheless, a majority of previous studies had attempted both segmentation accuracy and model efficiency individually. The current study addresses this gap by using YOLOv11 and RF-DETR on same dataset and hyperparameters to show that transformer based object detection models can equally perform on medical images.

## Materials and methods

**Fig 1:**
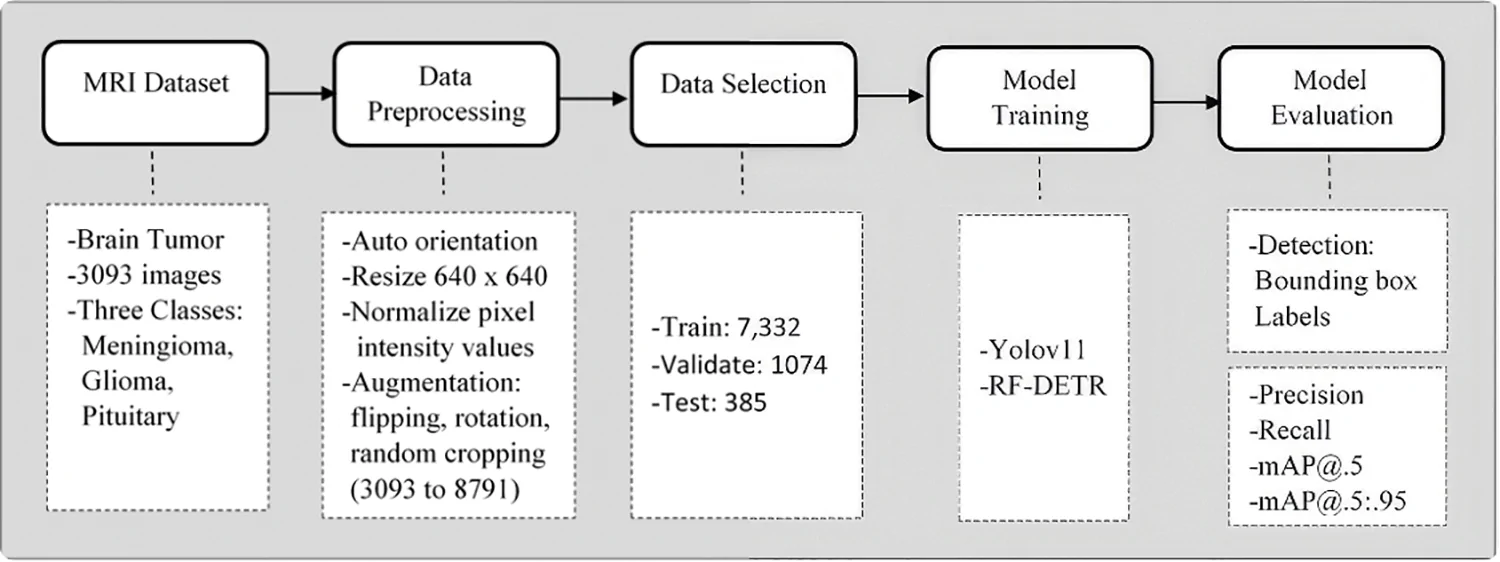
Workflow of brain tumor detection.

This section outlines the systematic steps developed for the detection and classification of brain tumors using advanced deep learning models i-e YOLOv11s and RF-DETR(small). Fig 1 shows summary of all the stages starting from data collection, pre-processing, data selection model training to model evaluation.

### Dataset description

The Roboflow’s Brain Tumor MRI dataset [21] is used for this study, a publicly accessible, human-labeled medical imaging dataset. The data set consists of 3093 MRI images and categories of clinically significant tissue, which normally consist of pituitary tumors, meningioma, glioma, and normal (no tumor) cases. Bounding boxes containing class and location of tumors are used to annotate images to provide localization training as well as classifications training to deep learning models.

Table 1 presents the class-wise distribution of images and annotated instances. Because datasets may contain multiple annotations per image, the summed class-wise image counts exceed the number of unique MRI images in the dataset. Moreover, instance counts provide a more representative measure of class distribution. Each instance corresponds to a labeled tumor region annotated with a bounding box, resulting in a total of 9,906 annotated instances across the four classes used for model training and evaluation.

**Table 1:** Per-class image count.

Tumor Class	Images	Instances
Glioma	2625	3026
Meningioma	2447	2500
No Tumor	1372	1372
Pituitary	2841	3008

### Data pre-processing and augmentation

For MRI image denoted by $I\in {\mathbb{R}}^{H\times W\times C}$, a geometric transformation was initially used in order to spatially align the images, *T*(*x*,*y*) so that there is a uniform anatomical orientation. Each picture was then resized to 640×640 pixels through the use of a stretch-resizing option to meet model input requirements. The pixel intensities were scaled using mean normalization of variances, $I_{n}=(I-\mu )/\sigma$, where μ and σ indicate the mean and standard deviation of the data. This pre-processing helped in having the same spatial dimensions and enhanced numerical stability in the optimization.

In order to increase the degree of generalization and reduce the effects of overfitting, stochastic augmentation has been used in the training process, where the total number of images has been increased to 8,791. For each training image $I_{n}$, geometric and spatial transformations were used to create three more variations. Horizontal flipping was characterized by $I_{hf}(x,y)=I_{n}(W-x,y)$, while fixed rotations of $\pm 90^{\circ }$ and small random rotations $\theta \in [-15^{\circ },15^{\circ }]$ were applied. The application of the rotation operator was used so that $I_{rot}(x,y)=I_{n}(R_{\theta }(x,y))$. Random zoom factor cropping, α∈[0,0.2], was expressed as $I_{crop}(x,y)=I_{n}(x+x_{0},y+y_{0})$. Training sample may thus be represented as the final sample of training, $I_{final}=A(I_{n})$, where A(·) represents a stochastic augmentation operator that is used only in training that enhances robustness to changes in the orientation, scale and acquisition settings.

### Loss functions

Both YOLOv11 and RF-DETR maximize a composite detection loss comprising of localization and classification losses:


$$\begin{array}{c} {ℒ}_{total}={ℒ}_{box}+{ℒ}_{cls} \end{array}$$ (1)

#### Box loss.

YOLOv11 uses an IoU (Intersection over Union)-based regression loss with Distribution Focal Loss (DFL) in order to estimate refined boundaries [22,23]:


$$\begin{array}{c} {ℒ}_{box}^{YOLO}=\lambda_{iou}(1-IoU(B_{p},B_{gt}))+\lambda_{dfl}{ℒ}_{dfl} \end{array}$$ (2)

$$\begin{array}{c} IoU=\frac{\left|B_{p}\cap B_{gt}\right|}{\left|B_{p}\cup B_{gt}\right|} \end{array}$$ (3)

RF-DETR, in its turn, is a variant of the DETR formulation [9], and it is the L1 regression with Generalized IoU (GIoU) loss [24].


$$\begin{array}{c} {ℒ}_{box}^{RF-DETR}=\lambda_{bbox}\|B_{p}-B_{gt}{\|}_{1}+\lambda_{giou}(1-GIoU(B_{p},B_{gt})) \end{array}$$ (4)

$$\begin{array}{c} GIoU=IoU-\frac{\left|C-(B_{p}\cup B_{gt})\right|}{\left|C\right|} \end{array}$$ (5)

where *C* denotes the smallest enclosing box covering prediction $B_{p}$ and ground truth $B_{gt}$. RF-DETR applies bipartite Hungarian matching prior to loss computation.

#### Classification loss.

YOLOv11 employs the use of Binary Cross-Entropy on a class-by-class basis [22].:


$$\begin{array}{c} {ℒ}_{cls}^{YOLO}=-\sum_{c=1}^{C}\left[y_{c}\log(p_{c})+(1-y_{c})\log(1-p_{c})\right] \end{array}$$ (6)

RF-DETR uses matched object query softmax Cross-Entropy [9]:


$$\begin{array}{c} {ℒ}_{cls}^{RF-DETR}=-\sum_{c=1}^{C}y_{c}\log(p_{c}) \end{array}$$ (7)

where *C* is the number of classes, *yc* is the ground-truth label, and *pc* is the predicted probability.

#### Distribution focal loss.

DFL, only applied to YOLOv11, is a discrete probability distribution modelling coordinate offsets to increase the localization accuracy [23]:


$$\begin{array}{c} {ℒ}_{dfl}=-\sum_{i=1}^{n}w_{i}\log(p_{i}) \end{array}$$ (8)

where *pi* represents predicted probabilities over discrete bins and *wi* are interpolation weights derived from ground-truth offsets.

RF-DETR does not use DFL, but it uses L1 and GIoU optimization in its end-to-end transformer.

### Training and testing strategy

The dataset was split into training, validation, and testing subsets to allow an unbiased evaluation. Data augmentation was applied exclusively to the training subset after the split was established, ensuring that the validation and testing subsets remained strictly independent and consisted only of original, unaltered images. The split is presented in Table 2, by using automated dataset versioning functions of Roboflow. It makes sure that adequate information is present to train the model and some samples are not used in model training so as to have sound validation and ultimate testing.

**Table 2:** Dataset splitting summary.

Dataset Distribution	Images	% of data
Training set	7,332	83%
Validation set	1,074	12%
Test set	385	4%

### Experimental parameters and computatuional complexity

The experiment uses GPU model RTX A6000, 49140 MiB, and the deep learning framework chooses Python-3.10.0 torch-2.1.1 + cu118 CUDA:0. The model’s hyperparameters are shown in Table 3.

**Table 3:** Hyperparmeters settings.

Hyperparameter	YOLOv11s	RF-DETR(small)
Input Resolution	640 x 640	640 x 640
Learning Rate	0.01	0.0001
Batch Size	32	16
Epoch	150	100
Weight Decay	0.0005	0.0001
IoU Threshold	0.7	–
GIoU Loss	–	2
GIoU Cost	–	2
Optimizer	Auto (Ultralytics)	AdamW
Random Seed	0	42

While Table 4 is given for detailing the parameter counts, computational complexity (GFLOPs) and inference latency (ms) evaluated at the hardware and software platform given above.

**Table 4:** Computational complexity.

Model	Parameters	GFLOPs	Latency
YOLOv11s	9.41M	21.3	3.3ms
RF-DETR(small)	27.2M	25.31	3.5ms

### Model training

This study assesses two recently developed object detection models, YOLOv11s and RF-DETR(small), in brain tumor localization and classification in MRI images. YOLOv11s uses its effective convolutional neural network (CNN) based dense prediction system that allows quick and accurate localization. While RF-DETR(small) uses transformer based global reasoning by object queries and attention systems. Using these models separately under the same experimental conditions, this work makes it possible to arrange a comparative study of convolutional and transformer based detection paradigms in the framework of medical image analysis.

Crucially, this comparative framework must account for distinct pre-training foundations and their corresponding domain gaps when transitioning to medical modalities. YOLOv11s relies on supervised weights pre-trained on the COCO dataset, optimizing it for distinct category boundaries in natural scenes. Consequently, it faces a steep semantic gap when adapting its convolutional bounds to grayscale, low-contrast, and texture-dense MRI structures. Conversely, RF-DETR(small) utilizes a DINOv2 (ViT-L/14) backbone. Because DINOv2 leverages large-scale self-supervised learning, it develops generalized, patch-level feature representations that inherently excel at capturing long-range dependencies and fine-grained spatial features.

Thus, the observed performance disparities reflect more than just structural architecture; they highlight a critical clinical deployment trade-off between the high-capacity, resource-intensive representations of self-supervised transformer backbones and the highly efficient but domain-sensitive boundaries of edge-optimized convolutional detectors.

The implementation, source code, and configuration files for the proposed brain tumor detection are publicly available for reproducibility at https://github.com/fadeem/Brain-Tumor-Detection

#### YOLOv11.

**Fig 2:**
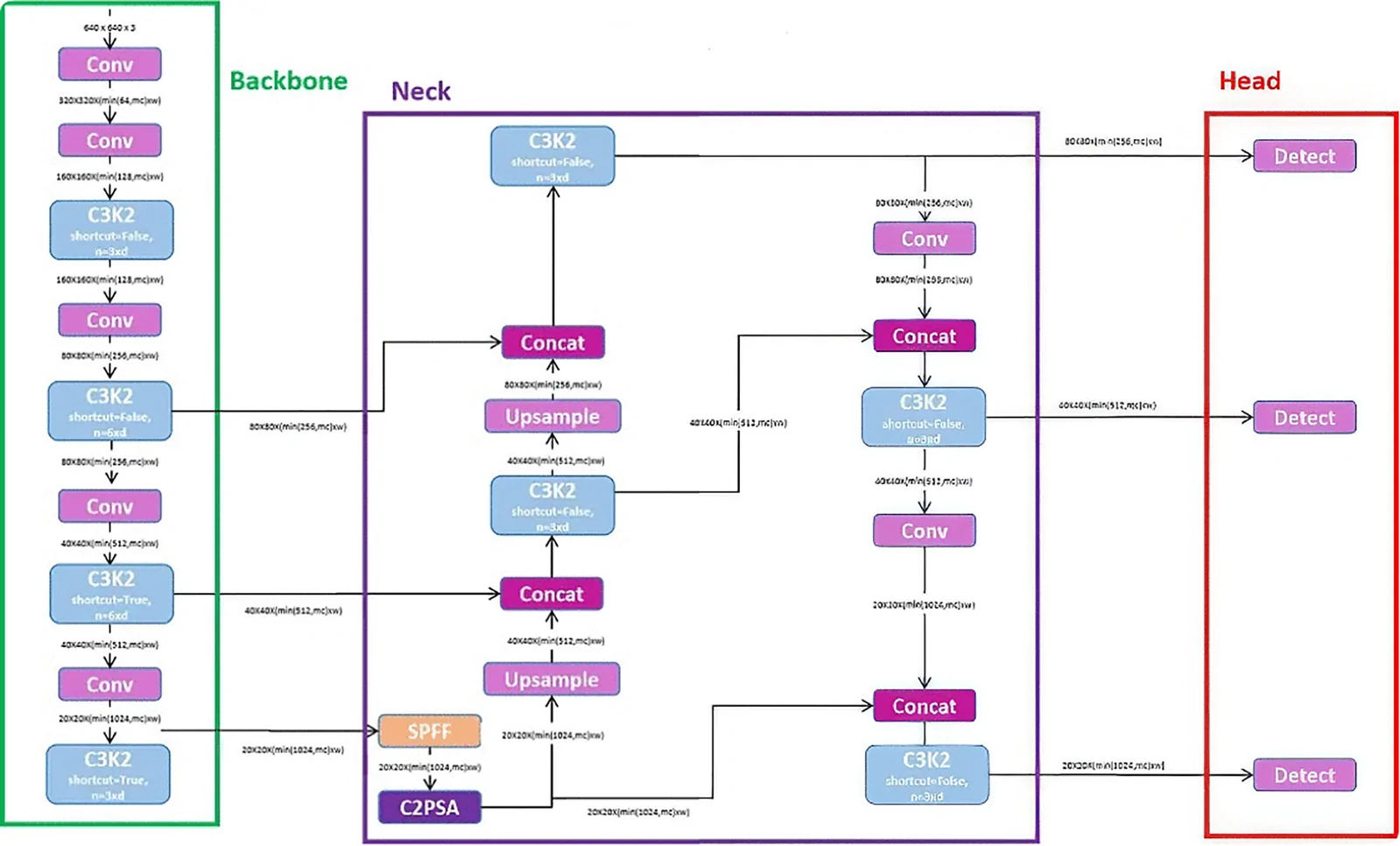
YOLOv11 architecture [25].

YOLOv11 [25] is a more recent development in the YOLO family of single-stage object detectors and is more focused on real-time inference and architectural efficiency. It poses object detection as a dense prediction problem, where the bounding boxes and the class probabilities are directly regressed using the spatial feature maps in one forward pass. YOLOv11s, which is initialized with pre-trained COCO weights and fine-tuned on the Brain Tumor MRI dataset, shows a high ability in the detection of small and irregular tumor regions. Its architecture is a backbone-neck-head architecture where hierarchical aspects are obtained through stacked convolutional layers, and aggregated through enhanced multi-scale feature fusion plans in order to enhance robustness in the context of different tumor sizes. The detection head does not use region proposals to conduct dense predictions, and thus facilitates localization and classification with efficiency. The general workflow of YOLOv11 can be summarized using Fig 2 below that emphasizes the successive stages of feature extraction, multi-scale fusion, and detection involved in the model.

#### RF-DETR.

**Fig 3:**
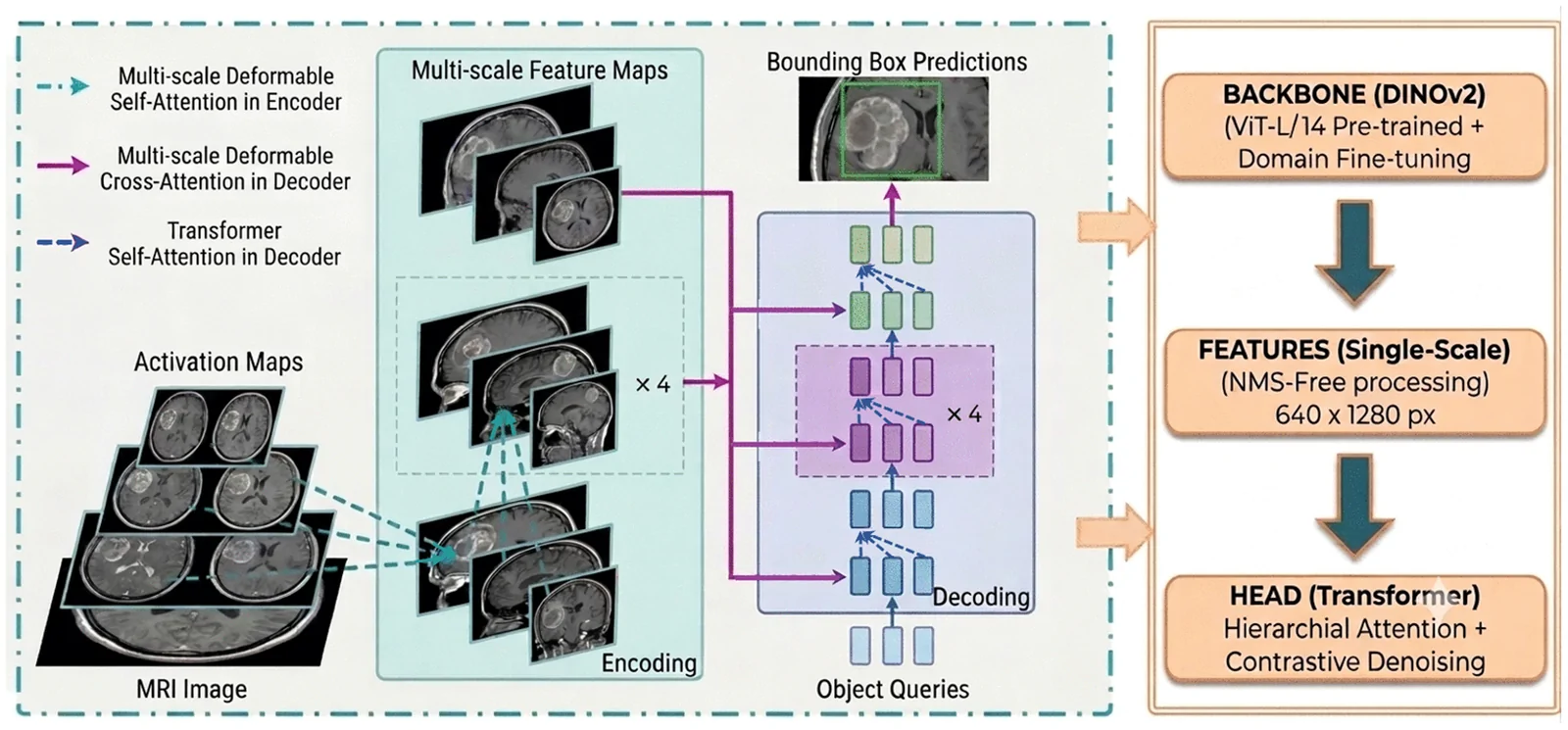
RF-DETR architecture, adapted from Zhu et al. [27] used under CC BY 4.0 license for illustrative purposes only.

RF-DETR [26] is a transformer object detector based on the DETR family, which aims to trade off between global context representation and real-time. In contrast to grid-based detectors, RF-DETR poses the problem of object detection in the form of a set prediction, omitting anchor boxes and non-maximum suppression. The model uses self-supervised DINOv2 vision transformer backbone which allows strong feature generalization and better responsiveness to the boundaries of diffuse tumors. In this paper, the Small version is used in order to have a good trade-off between detection accuracy and computational efficiency in medical imaging. RF-DETR architecturally computes single-scale feature representations and applies them to a transformer encoder-decoder with deformable attention thereby enabling efficient global reasoning at a lower computational cost. A fixed set of learnable queries generate the predictions of objects in parallel leading to consistent and stable detecting objects. RF-DETR inference operates in the following workflow, as illustrated in the Fig 3 below that identifies the sequential steps involved in feature extraction, encoding and decoding using transformers, as well as, query-driven prediction. Although the transformer architecture offers better global context modeling than the convolutional detectors, it is computationally more complex, and it is addressed by deformable attention and lightweight model versions.

### Evaluation parameters

Standard object detection metrics were used to measure performance of the model in terms of accuracy and robustness. The following parameters are used to get the detection efficacy based on Intersection over Union (IoU) thresholds:

- **Precision:** The precision of predicted bounding boxes is the ratio of the number of true positives (TP) to all positive predictions:

$$\begin{array}{c} \text{Precision}=\frac{TP}{TP+FP} \end{array}$$ (9)

- **Recall:** Measures how many ground-truth objects the model detects and it is the percentage of true positives to the total ground-truth objects.:

$$\begin{array}{c} \text{Recall}=\frac{TP}{TP+FN} \end{array}$$ (10)

- **Mean Average Precision (mAP):** As the main indicator, mAP calculates the average of the AP of all classes. AP is a summary of the precision-recall curve, which gives a global measure of the quality of detection at any confidence level.:

$$\begin{array}{c} \text{mAP}=\frac{1}{n}\sum_{i=1}^{n}AP_{i} \end{array}$$ (11)

The specific report of performance at mAP 0.5 gives standardized comparison and consistency in evaluation of reliability of the detection.

## Results

In this section, the performance of YOLOv11 and RF-DETR in terms of brain tumor detection is compared using detailed assessment measures based on the results of MRI. The main areas of model performance are included in the evaluation methodology. Both models were trained and tested on identical MRI brain tumor dataset (described in dataset section) with the same data division and evaluation procedures so that the models can be fairly compared. The standard metrics of object detection are used to determine performance, which are precision, recall, and mean Average Precision (mAP) at various IoU thresholds.

### Overall detection performance

Table 5 summarizes the overall detection performance of YOLOv11 and RF-DETR. The strengths of both detectors in each of the evaluation metrics are distinct. YOLOv11 has a higher mAP@.5 of 95,21% over RF-DETR, which has a 94.30% mAP@.5, meaning that YOLOv11 can detect slightly better at lower levels of IoU. This advantage is more evident at narrower localization metrics, with YOLOv11 achieving mAP@.5:.95 of 78.67% compared to RF-DETR, which scores 74.78% indicating that YOLOv11 is more resistant to the accurate localization of the bounding box. Regarding detection reliability, YOLOv11 is more accurate with 93.68 per cent than RF-DETR at 90.65%, this means that it is more efficient in suppressing false positives. Nevertheless, this should be paid with a price in the form of recall, with YOLOv11 performing much better than RF-DETR at 90.98% versus 85%, which is more capable of identifying actual tumor cases. On the whole, RF-DETR is more accurate, whereas YOLOv11 offers a balance between localization accuracy and complete tumor detection, which is more effectual in the case when the number of missed detections is critical.

**Table 5:** Overll performance.

Model	Precision%	Recall %	mAP@50%	mAP@50:95%
YOLOv11	93.6	91	95.2	78.7
RF-DETR	90.6	85	94.3	74.7

A limitation that we encountered due to severe computational constraints and the high resource demands of training and executing 3–5 independent runs for every configuration was not feasible. But in the modern object detection models like (YOLO and DETR architectures), training protocols are highly stabilized by large batch sizes, extensive pre-training (COCO weights), and long epoch schedules (e.g., 100–300 epochs), which naturally dampens random variance to negligible levels (usually <0.5%)

Further analysis is performed in detail to examines the model behavior in classes of tumors i-e Glioma, Meningioma, Pituitary, and No Tumor. Table 6 shows the precision, recall, and mAP@50:95 per-class by both models.

**Table 6:** Class-wise performance comparison between YOLOv11 and RF-DETR.

Class	Precision (%)		Recall (%)		mAP@50 (%)		mAP@50–95 (%)	
	YOLOv11	RF-DETR	YOLOv11	RF-DETR	YOLOv11	RF-DETR	YOLOv11	RF-DETR
Glioma	88.50	88.50	80.30	85.0	88.00	91.88	69.30	69.51
Meningioma	96.90	100	96.52	85.0	98.40	98.23	83.62	83.77
No Tumor	97.41	100	97.91	85.0	99.41	100	88.90	83.42
Pituitary	91.70	93.10	89.20	85.0	95.10	87.07	73.11	74.77

### Training and validation convergence behavior

**Fig 4:**
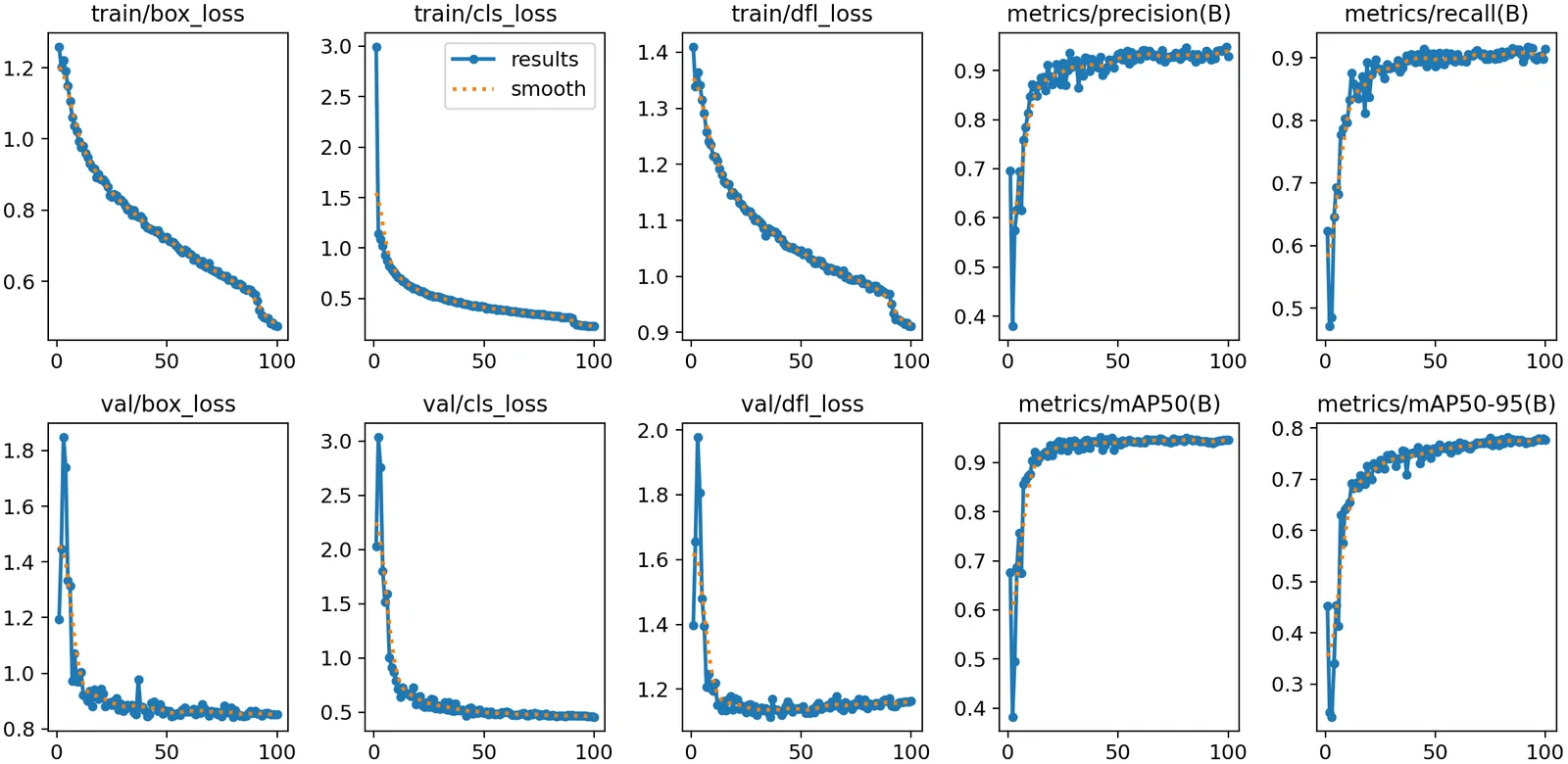
YOLOv11s performance.

**Fig 5:**
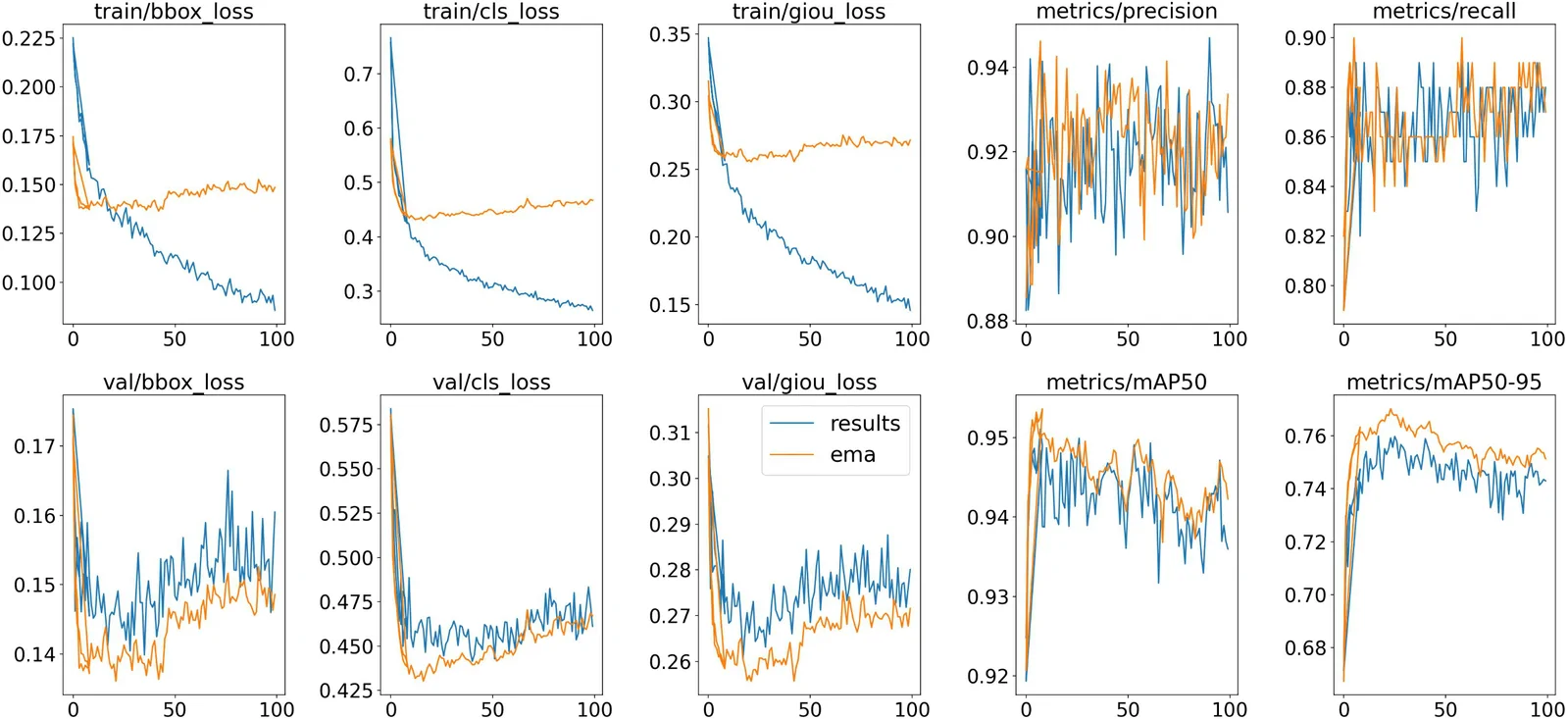
RF-DETR(small) performance.

The loss curves and metric trend across epochs are used to analyze the stability and convergence behavior of both models. YOLOv11s in Fig 4 shows smooth and monotonic convergence both in training and validation loss, leveling off around 100 epochs. Conversely, RF-DETR(small) exhibits more wavy curves of loss and metrics in early training, and the stabilization of the curves is slower, and it is observed to be stabilized at later epochs as indicated in Fig 5. This is explained by the transformer-based optimization process which uses global attention mechanism and set-based matching which results in a larger variance in the initial learning stages. Although it occurs at a slower convergence, RF-DETR(small) eventually attains competitive detection performance which suggests that its global reason ability offsets less stable initial training dynamics.

### Localization accuracy

The quality of localization is compared by examining the difference in the performance between mAP@50 and mAP@50:95 as indicated in Fig 4 and 5 of both YOLOv11s and RF-DETR(small). The smaller these metrics are, the more resilient it is to stricter IoU thresholds and less dependent on bounding box alignment. Table 7 shows that YOLOv11s has a localization gap of 16.5% with RF-DETR(small) having a gap of 19.6%, which indicates that the YOLOv11s exhibits more robust localization performance under stricter IoU thresholds. YOLOv11s uses box regression and Distribution Focal Loss (DFL) to increase the accuracy of boundaries whereas RF-DETR (small) uses L1 regression and Generalized IoU loss to optimize the position of the bounding box because their localization strategies are fundamentally different.

**Table 7:** Localization gap analysis.

Model	mAP@50	mAP@50:95	Localization Gap	Localization Ratio
YOLOv11s	95.2	78.7	16.5	0.83
RF-DETR(small)	94.3	74.7	19.6	0.79

These show that YOLOv11s is advantaged with its grid-based and dense predictions grid mechanism to perform fine-grained localization, and RF-DETR(small) focuses on global reasoning of objects, which might influence tight boundaries alignment in situations with high IoU requirements.

### Object detection analysis

**Fig 6:**
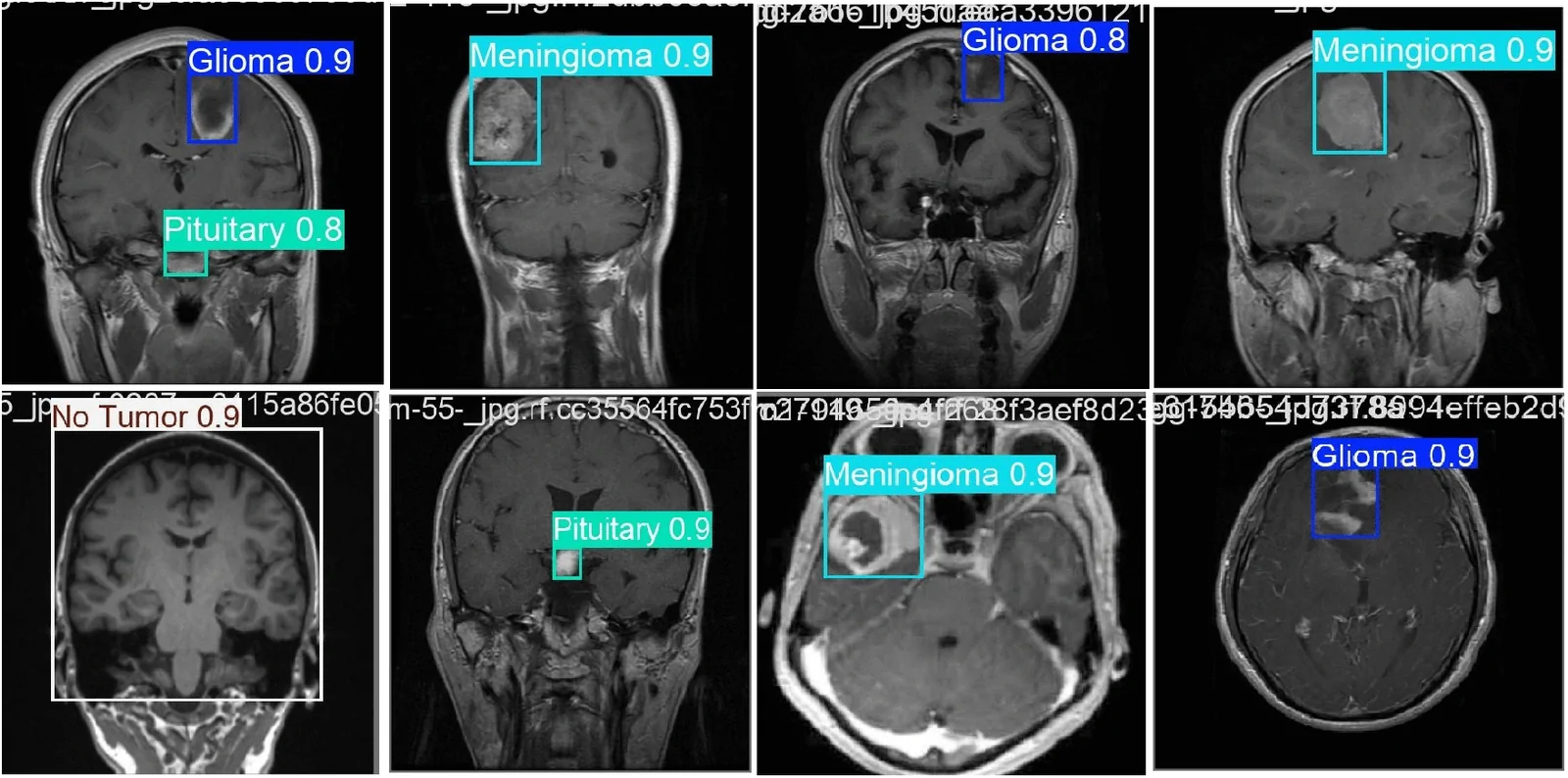
YOLOv11s prediction results. The underlying base MRI scans are obtained from the “Brain Tumor MRI” open-source dataset by workspace research-jz5sv [21] on Roboflow Universe, used under the CC BY 4.0 license.

**Fig 7:**
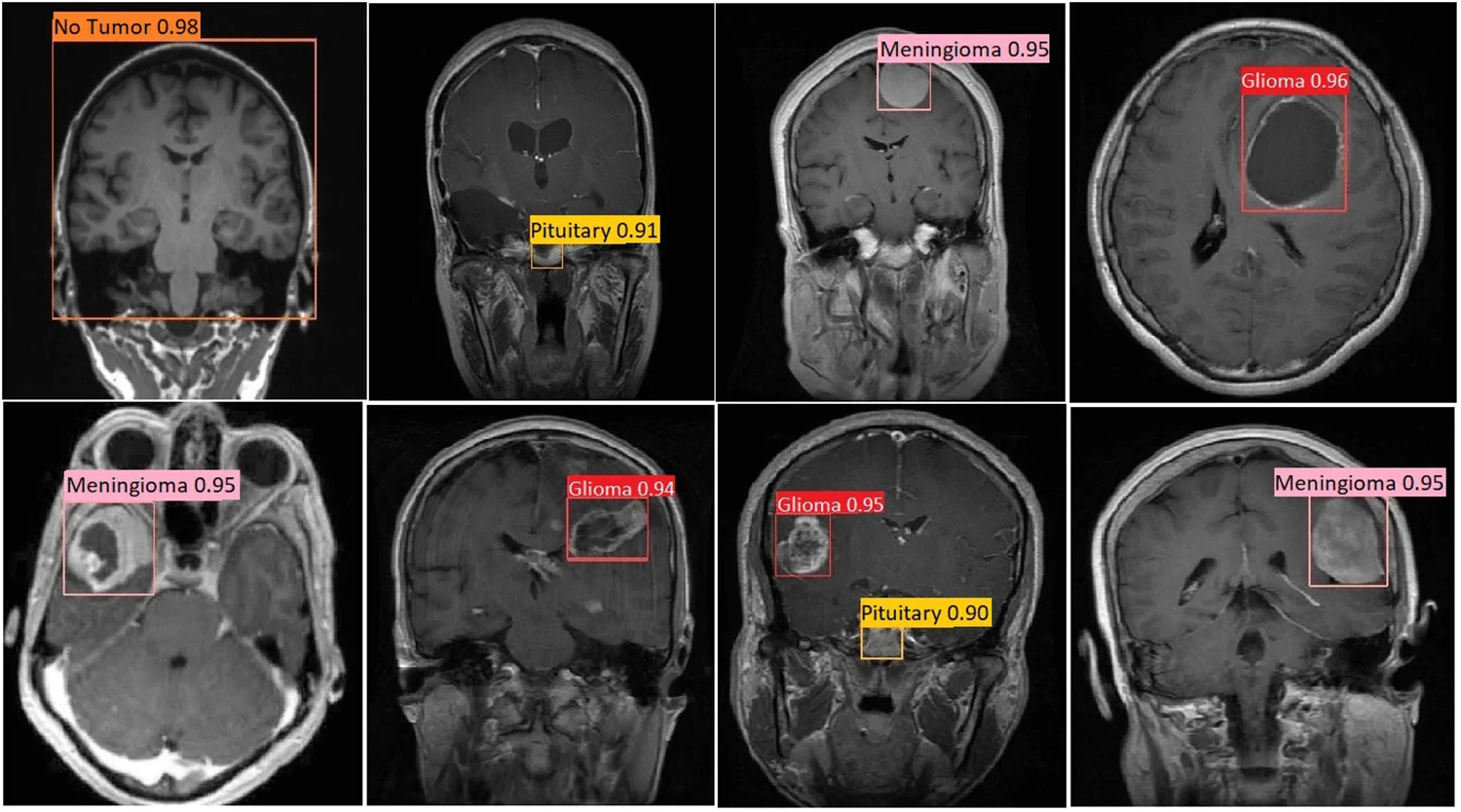
RF-DETR(small) prediction results. The underlying base MRI scans are obtained from the “Brain Tumor MRI” open-source dataset by workspace research-jz5sv [21] on Roboflow Universe, used under the CC BY 4.0 license.

Sample results of both models are provided in Fig 6 and Fig 7 giving the illustrations of the typical outputs of the detection of the two models. The dataset comprises four target classes—Glioma, Meningioma, Pituitary, and No Tumor. Following the annotation protocol provided with the dataset, each class, including the No Tumor category, is represented by a bounding box annotation. During inference, the detection models predict a bounding box, class label, and associated confidence score indicating the probability of the detected region belonging to the predicted class.

YOLOv11s makes correct and narrow detections on clear tumors, and has reduced false detection on clear areas. On the other hand RF-DETR(small) demonstrates a higher ability to detect tumors of diffuse border or low contrast, which is advantageous since it can model long-range dependencies throughout the image. Nevertheless, there are some cases of overestimation of bounding box size, which is also in line with its predictions that are driven by global attention. Such visual comparisons supplement the quantitative findings and offer some practical understanding on strengths and weaknesses of each method in clinical MRI analysis.

### Study limitations

There are a number of limitations to this study. Firstly, the evaluation was performed on a single public MRI dataset of brain tumors, which could reduce the generalizability of the results to images from other institutions, scanners, and patient cohorts. Second, there was no external validation using independent clinical data, which should be included in future studies to evaluate the model’s robustness in real-world applications. Third, the study only considered the image-based tumor detection and classification without using other clinical or pathological information that can improve the diagnostic performance. Lastly, like any supervised deep learning technique, the outcomes rely upon the quality and uniformity of the marked preparing information.

## Conclusion

The comparative analysis shows that YOLOv11s is outstanding in terms of stability in the training process, precision in localization, and computational efficiency, and it can be utilized in clinical settings with the constraint of resources or real-time requirements. RF-DETR(small) is more computationally expensive and slower to converge, but shows a high level of global contextual knowledge, and this can be useful in the detection of subtle or visual ambiguity tumors. In general, these findings indicate that CNN-based and transformer-based detectors complement each other. These factors should be used to determine which between YOLOv11s and RF-DETR(small) best fits the application, including the speed at which it can be used to make inferences, the localization accuracy, and the tolerance of training complexity.
